# An Evaluation of the Quality of Parent-Child Interactions in Vulnerable Families That Are Followed by Child Protective Services: A Latent Profile Analysis

**DOI:** 10.3390/children8100906

**Published:** 2021-10-11

**Authors:** Ananda Stuart, Catarina Canário, Orlanda Cruz

**Affiliations:** Faculty of Psychology and Education Science, University of Porto, 4200-135 Porto, Portugal; anacanario@fpce.up.pt (C.C.); orlanda@fpce.up.pt (O.C.)

**Keywords:** parent-child interaction, quality, child protective services, vulnerable families, observation, structured task, micro-analytic coding, discrete behaviors, global ratings, latent profile analysis

## Abstract

In the current study, an observational procedure, recorded in video, was used to evaluate the quality of parent-child interactions in a sample of vulnerable Portuguese families (*n* = 47) with school-aged children followed by Child Protective Services (CPS). The study sought to explore if the families presented different profiles of parent-child interaction quality, and to characterize such profiles in terms of discrete behaviors observed, parenting outcome variables, and families’ sociodemographic and CPS referral characteristics. The parent-child dyads took part in a 15 minutes structured task and parents completed self-report measures (affection, parenting behaviors, and stress). Discrete behaviors of parents and children during interactions were coded with a micro-analytic coding procedure. The global dimensions of the parents’ interactions were coded with a global rating system. A latent profile analysis, estimated with global dimensions, identified two subgroups, one subgroup in which parents displayed higher quality interactions (*n* = 12), and another subgroup in which parents displayed lower quality interactions (*n* = 35). Further analyses comparing the subgroups determined that the higher quality subgroup presented more positive behaviors, and the lower quality subgroup presented more negative behaviors during the interactions. No further differences or associations were found regarding the parenting outcome variables, and the families’ sociodemographic and CPS referral characteristics. The findings are in line with prior studies, suggesting that vulnerable families may frequently present depleted parent-child interactions. However, given the small sample size, future studies should replicate the described procedures and analyses in larger sample sizes.

## 1. Introduction

Previous research has shown that the quality of a parent-child relationship has important consequences for a child’s development. Specifically, different studies have addressed the characteristics of adequate family interactions, as well as those characteristics with the potential to jeopardize children’s development [1,2,3,4]. Research findings in the field of parent-child relationships and interactions are very relevant, since the family has been acknowledged as one of the first socialization environments for each individual [5,6,7]. Parent–child interactions are, thus, determinants not only for the development of children, but also for the wellbeing of families. These interactions are modeled by the social, emotional, and cognitive skills of both a parent and a child, in which they respond and adapt to each other’s style [4,8].

The parent-child relationship can be affected by numerous risk factors that hinder the quality of the relationship, such as psychosocial and sociodemographic aspects which are well described in the literature [9]. These risk factors, in turn, play a negative role in short- and long-term child psychological wellbeing [9,10]. Low quality parent-child interactions, such as those experienced by abused and neglected children, have the potential to jeopardize children’s development, including their attachment and socio-emotional development, as well as lead to behavioral problems [11,12,13].

Child maltreatment behaviors are not isolated incidents, but rather occur as part of ongoing patterns of interactions that make up the parent-child relationship [14]. Abusive parents are thought to be inconsistent and ineffective in gaining their children’s compliance; hence, they resort to aversive behaviors (e.g., yelling and negative physical touch) more quickly, and reciprocate their children’s aversive responses more than non-maltreating parents. Other studies have established that parents with negative interactions, but not an extreme form of maltreatment, also have inconsistent and low-warmth parenting, but not continuously [15]. Furthermore, they can also be influential in the development of emotional and behavioral challenges in children and adolescents, including conduct disorder [16].

Data from the 2020 annual report from the Portuguese Child Protective Services (CPS) [17] detail the sociodemographic aspects of children followed by the services, and who they live with, including age, sex, education levels, and income of the main caregiver. Moreover, it describes reasons that have led to a referral to CPS, with most of the reasons, in Portugal, being exposure to domestic violence, followed by neglect. Over 80% of the families followed by CPS are referred to parenting support in community-based services. However, less exhaustively investigated are the parent-child relationships and quality interactions in the families followed by CPS and referred to parenting support, considering the evidence suggesting that dysfunctional parent-child interaction dynamics are strong predictors of child maltreatment [8,18].

Since the 1980s, several intervention projects have incorporated information on parent-child interactions, with interventions focused on parent-child relationships directly integrated into the intervention design [19,20,21]. However, since the 1990s, there has been a more extensive recognition that research on these relationships may have critical implications for designing interventions [22], as can be seen in a meta-analysis by Wilson and colleagues [23]. In this meta-analysis, the researchers concluded that maltreating caregivers showed higher levels of aversiveness (e.g., rejection and reactive behaviors) and lower levels of positive and involvement interactions (e.g., praise and affectivity) in parent-child interactions than non-maltreating caregivers. Therefore, the improvement of parent-child interaction quality is a major goal of intervention programs [1].

Past research has shown that maltreating families were frequently characterized by lasting dysfunctional parent-child interactions in which a parent showed unpredictable, hostile, rejecting, and/or unresponsive behavior towards their child [13,24,25]. Abusive parents tend to be more reactive, from a psychophysiological point of view, to children’s aversive behaviors. In addition, they use more coercive discipline, have unrealistic expectations of the children, interact less with their children, are more negative than positive during interactions, and see their children as problems or as intentionally acting in an annoying manner [26]. As previously stated, parent-child interactions are crucial for children’s development and adjustment. When provided by parents with feelings of security, parent-child interactions foster adequate children’s development [1,27]. In addition, high-quality parenting interactions have a long-lasting impact on children’s abilities to thrive in academic settings. For example, cognitive functioning is significantly influenced by parenting behaviors, such as talking to and reading to their children [16,28]. Thus, to be able to develop suitable methods for strengthening the parent-child relationship, it is crucial to properly evaluate the quality of the interaction, using sound tools and procedures.

Parents play a decisive role in the socio-emotional development of children, and therefore direct behavioral observation has been considered to be the gold standard method for parent-child interaction assessment [29]. In addition, it should be highlighted that direct behavioral observation is the most effective method to evaluate parent-child interactions. Such a procedure has advantages over other more common methods used in clinical settings, such as self-report measures and structured interviews [29]. Advantages include the ability to generate data beyond what can be obtained by self-report, high clinical usefulness for professional decision making, and sensitivity to intervention-induced changes [30,31]. The implementation of behavioral observation methods in clinical practice can also allow professionals to observe infrequently occurring behaviors, analyze the antecedents or consequences of the behaviors, and register the complex aspects of social interactions [32].

Despite the relevance of the observational procedures to address parent-child interactions, these procedures are often underused in studies. In the current study, an observational procedure using a video-recorded structured task was used to address the quality of parent-child interactions in a sample of vulnerable families with school-aged children followed by CPS. The study had the following objectives: (1) to explore if the families presented different profiles of quality in their interactions, (2) to characterize such profiles in terms of the discrete behaviors observed, (3) to characterize such profiles in terms of a parent’s communication and affection, criticism and rejection, positive parenting, poor monitoring subscales, and parenting stress, and (4) to characterize such profiles in terms of the families’ sociodemographic and CPS referral characteristics.

## 2. Materials and Methods

### 2.1. Participants

The participants were recruited from families with at least one 6-to-12-year-old child, followed by CPS, and referred to parent support, in the district of Porto, Portugal. Exclusion criteria were applied when (a) the child or the parent suffered from developmental disorders, severe cognitive disability, or severe mental disorder; (b) the parent was recovering from drug addiction in the past year; or (c) the child or the parents were unable to understand European Portuguese. 

A total of 47 parent-child dyads completed the evaluation procedure. Participants were mostly mothers (95.7%), with ages ranging from 22 to 59 years old (*M* = 35.91, *SD* = 7.08) and the number of children was from 1 to 6 (*M* = 2.70, *SD* = 1.16), unemployed (55.30%), on average, with 7.68 years of schooling (*SD* = 3.41), from urban areas (68.10%), and without prior contact with positive parenting programs (87.20%). The children were mostly male (*n* = 29, 61.70%), attending the first cycle of basic education (63.90%), with ages ranging from 6 to 12 years old (*M* = 8.91, *SD* = 1.85). Most of the children were living in single-parent (46.8%) or reconstituted (27.70%) family households, followed by CPS due to neglect (40.40%) or exposure to domestic violence (25.50%), and about half of the children had prior referrals (at least one) to CPS (42.60%), in their lifetime.

### 2.2. Procedure

The current study is part of a wider research project aimed to evaluate the effectiveness of the Standard Triple P (STP) individual format parent program, when delivered to vulnerable families followed by CPS and referred to parenting support in community-based services (for further details on the REUNIRmais project, see Canário and colleagues [33]). The REUNIRmais project received ethical approval from the Ethics Committee of the Faculty of Psychology and Education Science of the University of Porto (approved 14 April 2020, reference 2020/04-2), and approval from the Data Protection Unit of the University of Porto (approved 03 September 2019, reference 2018091915006231).

The current study presents baseline data from the REUNIRmais project, in which the parents completed different measures in the presence of researchers, and each parent-child dyad took part in a structured interaction activity. For the purpose of the current study, in addition to the structured interaction activity, data regarding the measures completed by parents is presented. All families provided informed consent and signed release forms authorizing both the parent’s and child’s participation in the study. The child’s verbal assent was also obtained.

### 2.3. Measures 

**Sociodemographic Questionnaire.** A questionnaire was developed to collect data on the family and household’s characteristics (e.g., age, years of schooling, marital status, occupational status, and social support), and the child’s characteristics, including difficulties and concerns regarding their behavior. Information on the reasons for a family’s referral to CPS were provided by the CPS professionals. 

**Alabama Parenting Questionnaire (APQ)** [34]. The APQ Portuguese version [35] was used to assess parenting practices. It contains 42 items rated on a 5-point Likert scale based on a parent’s appraisal of their behavior frequency, ranging from 1 (“never”) to 5 (“always”). The study of the psychometric properties of the APQ Portuguese version [31] identified adequate psychometric properties of a three-factor model (positive parenting, ineffective parenting, and poor monitoring) in a solution of 20 items. For the first subscale, higher scores indicate better parenting strategies, whereas for the other subscale, higher scores indicate worse parenting behaviors. In the current study, the reliability coefficients were deemed to be acceptable for positive parenting (0.83) and for poor monitoring (0.72), but not for ineffective parenting (0.54). As such, ineffective parenting was not included as an outcome variable in the current study. 

**Affection Scale (AS)** [36]. This scale evaluates the parent-child relationship through two subscales that address the affection-communication and criticism-rejection of the parent towards their child, respectively. The scale has two versions: one to evaluate the parent’s perceptions of affection-communication and criticism-rejection in their relationship with their child, and another to evaluate the child’s perceptions of affection-communication and criticism-rejection in their parent relationship and interactions with their parent. In the current study, the first version was used. The scale includes 20 items rated on a 5-point Likert scale (from 1 “never” to 5 “always”), and a global score for each subscale is obtained by summing the answers to the items. Higher scores reveal higher levels of affection-communication and criticism-rejection. In the current study, the reliability coefficients for the affection-communication and criticism-rejection subscales were 0.73 and 0.76, respectively. 

**Parental Stress Index Short Form (PSI-SF)** [37]. The Portuguese version of the PSI-SF [38] was used to assess parental stress. The measure consists of 36 items rated from 1 (“strongly disagree”) to 5 (“strongly agree”). As in its original version, the Portuguese version of the measure provides three subscales, each with 12 items: (1) parental distress, (2) parent-child dysfunctional interaction, and (3) difficult child. In addition, a global parental stress score is obtained by summing the answers to the 36 items. In the global score scale, as in all subscales, lower scores indicate more difficulties and higher levels of stress. In the current study, only the global parental stress score was used, and the reliability coefficient was 0.92.

**Parent–child Interaction.** Each parent-child dyad took part in a structured task. The activity was developed in a separate room by the parent-child dyad and video recorded. The structured activity consisted of a jigsaw puzzle, with 48 or 64 pieces, depending on the child’s developmental level (children up to 9 years old used one with 48 pieces, and those above 9 years old used one with 64 pieces). Each dyad would sit at a desk in the room without other stimuli (e.g., toys), with the parent and child sitting next to each other and facing the camera. The researcher would place the puzzle pieces face up on the desk and provide an A5 picture of the complete puzzle. Then, the researcher would ask the parent to get the child to complete the puzzle, set up the camera to record the activity, and leave the room. The activity was programmed to be completed in 15 min. After this period, the researcher would see if the dyad was still performing the activity. If needed, further time was provided to the dyad to complete the activity. However, when the dyad took more than 15 min to complete the activity, only the first 15 min of the video were considered for coding purposes. The interactions lasted between 6 and 15 min, and the mean length was 12.56 minutes (*SD* = 2.90). 

Observations of the parent-child interactions during the structured task were coded following two coding systems. The first coding system consisted of a micro-analytic coding procedure adapted from the Family Observation Schedule (FOS) [39]. The FOS is a micro-analytic coding system, found to be reliable and sensitive [40], in which the presence or absence of behaviors of both the child and the parent under observation is scored in 10 s intervals. In the current study, the observation interval was adapted to a 30 s period. Further adaptations to the system excluded two discrete behaviors regarding parents (contact and aversive contact) that were not identified during the evaluations of the videos. Moreover, their definition was very similar to two more comprehensive behaviors that were maintained (social attention and aversive social attention). In addition, following a deductive approach, three additional discrete behaviors were included regarding parents’ behaviors (performs side-by-side with the child, and performs preventing the child from performing), and child behaviors (noncompliance). All three additional discrete behaviors were observed in the pilot study. The discrete behaviors used in the micro-analytic coding procedure are described in Table 1. Whenever the parents took less than 15 min to complete the structured task, to prevent possible scoring bias related to the intervals considered in the observation, a prorating approach was considered to estimate the number of behaviors per 15 min of interaction (30 intervals).

The second coding system consisted of global ratings of the following dimensions: involvement, positive affectivity, responsiveness, directivity, and stimulus quality. These global ratings were developed following an inductive and deductive approach on the basis of theoretical and empirical considerations, including the parents’ behaviors identified in preliminary data analysis conducted using a small pilot sample. Each parent was scored on each dimension on a 5-point Likert scale: 0 (“Never”), 1 (“Rarely”), 2 (“Half the time”), 3 (“Frequently”), and 4 (“Very frequently”). The dimensions used in the global ratings are described in Table 2. 

Data were coded by a trained psychology researcher following the two coding systems. To ensure the reliability of the coding, a second independent researcher also coded 36.17% of the interactions (*n* = 17). The inter-rater agreement revealed adequate values, with the discrete behaviors ICC ranging from 0.00 to 1.00 (*M* = 0.71), and the global ratings κ ranging from 0.72 to 0.90 (*M* = 0.77).

### 2.4. Data Analysis

The descriptive statistics for each outcome variable (mean, standard deviation, kurtosis, and skewness) were estimated using the software IBM SPSS (v0.27) [41].

Using the global ratings, latent profile analyses (LPAs) were performed to identify homogeneous subgroups within the sample. The profiles were estimated using the TIDYLPA CRAN package [42] in the R Studio software version 1.4.1103. Different models were estimated to identify the best fit to the data. Model fit was evaluated considering the Akaike’s information criterion (AIC), the approximate weight of evidence (AWE), the Bayesian information criterion (BIC), the classification likelihood criterion (CLC) and the Kullback information criterion (KIC), in which lower values indicate a better fit, and entropy, in which values above 0.64 are deemed to be acceptable [43]. The model fit comparisons were performed according to the guidelines defined by Akogul and Erisoglu [43] in which the lower AIC, AWE, BIC, CLC, and KIC values indicate a better fit to the data. For the current study, no power analysis is presented regarding the LPAs. As acknowledged by Spurk and colleagues [44], power analysis regarding latent profile analysis cannot be presented in every study because of their complexity. LPAs’ power analyses are made through simulation studies that require knowledge of population parameter values driven from prior work or theory, which are often unavailable [43].

Following the identification of two classes (profiles), independent sample *t*-tests were performed to evaluate the differences between classes regarding the discrete behaviors evaluated in the micro-analytic coding procedure, as well as regarding the scores of the communication-affection, criticism-rejection, positive parenting, and poor monitoring subscales, and the parenting stress index score, and continuous sociodemographic variables (parents’ ages, number of children, and years of schooling, children’s ages, and years of schooling). The independent sample *t*-tests were performed using the software IBM SPSS (v0.27) [41]. A priori power analysis performed using the software G*Power version 3.1.9.7 [45,46], deemed the sample size to be adequate to identify large effect sizes (Cohen’s *d* = 1.3) when considering two samples (allocation ration *N*_2_/*N*_1_ = 3, *N*_2_ = 33, *N*_1_ = 11, total *n* required = 44), *α* = 0.05, *power* = 0. The Cohen’s effect size d is reported in each *t*-test performed, and should be interpreted according to Cohen’s [47] guidelines, in which 0.20 regards a small, 0.50 a moderate, and 0.80 a large effect size. A Chi-square association test was also performed to further explore the associations between the classes and nominal sociodemographic- (parents’ employment status, and area of living, and children’s sex and family type) and CPS referral-related (reason for referral and any prior referral) variables.

## 3. Results

### 3.1. Preliminary Results

The descriptive statistics (mean, standard deviation, kurtosis, and skewness) of the discrete behaviors and global ratings coded from the observations of the parent-child interactions are presented in Table 3. The variables all seem to have a normal distribution, except for the parents’ discrete behaviors of aversive question and no interaction (where the scores were 0 for 45 and 38 parents, respectively), and for children’s discrete behaviors of aggression, opposing behavior, interruption, and no interaction (where the scores were 0 for 46, 47, 31, and 44 children, respectively). Due to the identified distribution problems, the above-mentioned variables were not used in the subsequent analyses. The outcomes provided by the measures present normal distributions.

### 3.2. Latent Profile Analysis

**Figure 1:**
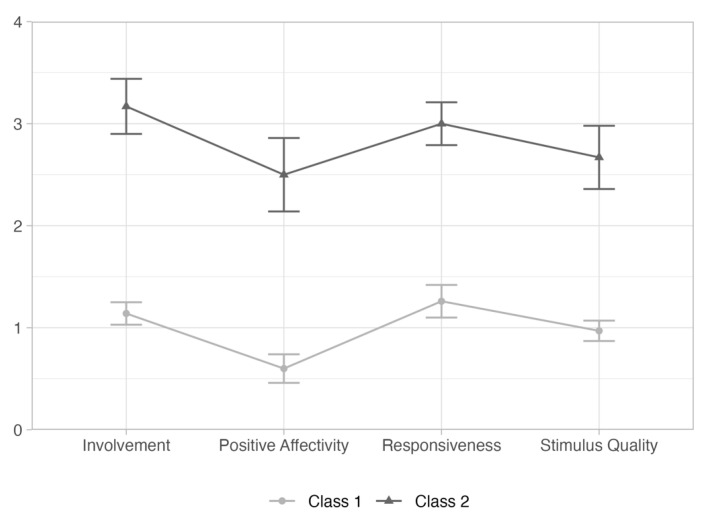
Latent profile analysis of the global rating dimensions.

Different LPA models were estimated. The first model included the five global rating dimensions (involvement, positive affectivity, directivity, responsiveness, and stimulus quality) as indicators to estimate one single class (profile) (AIC = 721, AWE = 806, BIC = 740, CLC = 703, and KIC = 734). The second model included the five dimensions as indicators to estimate two classes (profiles) (AIC = 622, AWE = 760, BIC = 652, CLC = 592, and KIC = 641), and was found to reveal a better fit to the data than the single class model. However, in the second model tested, the global rating dimension directivity did not contribute to differentiate the classes. Thus, a third model was tested including four global rating dimensions (involvement, positive affectivity, responsiveness, and stimulus quality) as indicators to estimate two classes (profiles). The third model revealed the best fit to the data (AIC = 462, AWE = 573, BIC = 486, CLC = 438, KIC = 487, and entropy = 0.99) and identified two classes (depicted in Figure 1). Class 1 included 35 participants who displayed a lower quality interaction with their child, characterized by less involvement, positive affectivity, responsiveness, and stimulus quality. Class 2 included 12 participants who displayed a higher quality interaction with their child, characterized by more involvement, positive affectivity, responsiveness, and stimulus quality. The estimates and standard error for the means and variances from the latent profile analysis are presented in Table 4.

### 3.3. Differences between Classes Regarding the Discrete Behaviors Evaluated in the Micro-Analytic Coding Procedure

Means and standard deviations for the discrete behaviors evaluated in the micro-analytic coding procedure by class (lower vs. higher quality interactions), as well as the values for the *t*-test, *p*-values, and Cohen’s d effect size are described in Table 5. Large effect size differences were found regarding praise, specific and vague instructions, question, social attention, and parallel performance; moderate effect size differences were found for aversive vague instruction and aversive social attention. On the one hand, parents who exhibited a higher quality interaction with their children praised their children, gave more instructions (both specific and vague), questioned more often, and paid more social attention than the parents who exhibited a lower quality interaction. On the other hand, parents who exhibited a lower quality interaction gave more aversive vague instructions, paid more aversive social attention, and more often performed the task side-by-side with their children than those who exhibited a higher quality interaction.

### 3.4. Differences between Classes Regarding the Measures Outcome Variables

Means and standard deviations for the scores of the parenting outcome variables obtained through the parents’ self-report measures on affection (communication-affection and criticism-rejection) parenting behaviors (positive parenting and poor monitoring subscales), and the parenting stress index by class (lower vs. higher quality interaction), as well as the values for the *t*-test, *p*-values, and Cohen’s d effect size are described in Table 6. Statistical differences were found between classes regarding the measured outcome variables.

### 3.5. Differences and Associations between Classes and the Families’ Sociodemographic and CPS Referral Characteristics

Means and standard deviations for the scores of continuous sociodemographic variables (parents’ age, number of children, and years of schooling, children’s age and years of schooling) by class (lower vs. higher quality interaction), as well as the values for the *t*-test, *p*-values, and Cohen’s d effect size are described in Table 7. Statistical differences were found between classes regarding the continuous sociodemographic variables.

Likewise, there were no associations between the classes and the nominal sociodemographic and CPS referral related variables. Specifically, there were no associations found between the classes and parents’ employment statuses (Fisher’s exact test = 0.74) and area of living (*χ^2^*(2) = 2.17, *p* = 0.34); between children’s sex (Fisher’s exact test = 1) and family type (*χ^2^*(2) = 3.26, *p* = 0.20); or between the classes and the reasons for referral (*χ^2^*(3) = 1.66, *p* = 0.65) and prior CPS referral (Fisher’s exact test = 1).

## 4. Discussion

The current study addressed the quality of parent-child interactions in a sample of vulnerable families followed by CPS. Specifically, the study sought to explore if the families presented different profiles of quality in their parent-child interactions, and to characterize such profiles in terms of discrete behaviors observed in the interaction, and also regarding parenting outcome variables, and the families’ sociodemographic and CPS referral characteristics.

Through an observational procedure of a structured task performed by parent-child dyads and video recorded, discrete behaviors by both parents and children, and global dimensions were coded through micro-analytic and global ratings coding systems, respectively. The reliability analysis, considering the inter-rater agreement, yielded adequate results. Among the total behaviors identified by the micro-analytic coding procedure, 18 behaviors revealed at least a moderate agreement, as indicated by the ICC values. Discrete behaviors such as aggression and opposing behavior, which revealed poor inter-rater agreements, were hardly ever identified in the current study. The literature suggests that in observed structured activities, especially in the context of play such as assembling a jigsaw puzzle, some behaviors such as aggression and opposing behavior tend to be displayed less often [48]. In the current study, children’s aggression and opposing behaviors occurred less often, which may have hindered the estimation of the inter-rater agreement, contributing to the poor agreement outcomes found. In addition, the agreement given by the κ coefficient revealed an almost perfect agreement for the dimension involvement, while for the other dimensions, it revealed a moderate agreement. The behavioral observation coding systems described in the current study seem to be a reliable and sound tool to evaluate the quality of parent-child interactions.

The LPA including four global rating dimensions (involvement, positive affectivity, responsiveness, and stimulus quality) enabled the identification of two homogeneous subgroups within the sample, one subgroup that including parents who displayed lower quality interactions (*n* = 35), and another subgroup that included parents who displayed higher quality interactions (*n =* 12) with their children. The result regarding the lower quality subgroup is in line with prior findings suggesting that, in at-risk families who experienced adverse environmental conditions, the parent-child interactions usually showed lower levels of positive interactions, responsiveness, sensitivity, and involvement [14,41]. Importantly, it should be highlighted that the current study’s results enabled the identification of a subgroup with higher quality interactions, even if it was in a smaller number of families. However, it should be noted that the global rating dimension of directivity was not considered in the characterization of parents into classes. The literature regarding parent-child interactions suggests that effective interactions are characterized by high responsiveness and moderate to low directivity [49]. Still, the parents in the current study presented a somewhat constant level of directivity, which precluded the dimension of contributing to the subgroups’ identifications.

Following the LPA, the subgroups were compared regarding the discrete behaviors identified through the micro-analytic coding system. The moderate to large effect size results revealed that positive discrete behaviors (praise, instructions, question, and social attention) were more frequent in the higher quality subgroup, whereas negative discrete behaviors (aversive vague instructions and performs side-by-side with the child) were more frequent in the lower quality subgroup. None of the children’s behaviors were different between subgroups, which is a somewhat different finding from that of other studies, in which negative parental behavior may lead to more externalizing problems in children [8,50]. Although child misbehaviors are a common topic of study in the area of child maltreatment, often these behaviors are more related to a parent’s perceptions and, indeed, do not represent their child’s behaviors displayed in the interaction [51]. Nevertheless, the lack of differences in the children’s behaviors between the subgroups can also be a consequence of the type of activity and method chosen, in which the nature of the observational structured activity does not allow for the variability of the child’s behavior over the interaction [48,52].

The subgroups were also compared regarding parenting outcome measures obtained through self-report questionnaires completed by the parents (communication-affection, criticism-rejection, positive parenting, poor monitoring subscales, and parenting stress). There were no differences found between groups regarding the parenting outcome variables which can be explained by the literature demonstrating that parents’ self-report measures only modestly correlate with observational measures [53,54]. It is possible that the parents’ self-report measures and the observational procedure address distinctive phenomena. The parenting outcome measures address global parenting constructs that are evaluated through questionnaires and regard parents’ behaviors and perceptions related to different situations of parents’ and children’s everyday lives, whereas the structured activity, herein evaluated, pertains to a specific situation in a given setting. Moreover, the observation procedure captures a parent’s behavior at a single time point (e.g., a 15 min interval), whereas the self-report measures require parents to reflect on their own behaviors over a longer period (e.g., over the last week, or the last month) and over several situations [55,56]. There were no differences or associations found for the classes identified regarding the families’ sociodemographic and CPS referral characteristics, which may be explained by the fact that the sample presents relatively homogeneous characteristics.

The procedures described in the current study regarding the observation and evaluation of parent-child interactions seem to not overlap with the results of the evaluation of the parenting outcome measures obtained through questionnaires. The observation and evaluation of a parent-child interaction appears to have its own specificity, and, as such, it may be a relevant tool when evaluating the needs of and results of parenting intervention programs, complementing the information obtained through questionnaires and interviews.

The current study has some methodological strengths that should be highlighted as follows: First, the use of different measures to thoroughly characterize the families, including two observation systems (micro-analytic coding and global ratings) and the use of valid self-reported measures; second, good levels of inter-rater agreement were found for the majority of parent’s and child’s behaviors, as well as for the global ratings’ dimensions; third, even though several studies have addressed parent-child interactions, few studies have included a sample of vulnerable families followed by CPS; fourth, most of the studies in the literature have focused on sociodemographic aspects, rather than on the behavioral patterns of parent-child relationships, which was the main objective of the current study.

Despite these strengths, some limitations should also be mentioned. This study has a small sample size. Data collection took place since October 2019 and was negatively affected by the lockdown measures imposed by the Portuguese Government to contain the COVID-19 pandemic, which precluded families’ face-to-face evaluations for several months, thus, limiting the ability of the research team to increase the sample size. Additionally, the observations of parent-child interactions were carried out in a single period, which may have contributed to a loss of external validity of the results from the observation measures. The small sample size is also acknowledged as a possible limitation to the LPA performed. As previously reported, no power analysis for LPA was estimated, as this procedure is identified in the literature as being complex and requiring knowledge of population parameter values which may not be available [44]. However, a relevant issue to address when performing an LPA is the parsimony and meaningfulness of the profiles. The subgroups, herein described, are meaningful and both subgroups regard more than 1% of the current sample (lower quality subgroup 74.50%, and higher quality subgroup 25.50%), even though the higher quality subgroup includes fewer than 25 participants [44]. As highlighted by Nylund-Gibson and Choi [57], smaller samples may be adequate with simpler models, with few indicators and classes, as is the case of the current study.

Considering the limitations discussed, specifically regarding the small sample size, the results should be interpreted with caution. Future studies should replicate the profile solution herein described with larger sample sizes. In addition, future studies should evaluate parent-child interactions at different periods of time (rather than a single time period), and should also evaluate the parent-child interaction in dyads from a community sample not followed by CPS, to further characterize and understand the contributions of family vulnerability and adversity to the quality of parent-child interactions.

## Figures and Tables

**Table 1:** Discrete behaviors used in the micro-analytic coding adapted from the FOS (Family Observation Schedule) and developed from a deductive approach based on the literature.

Discrete Behaviors	Description
*Parent Behavior Codes*	
Praise	A positive global reference to the child.
Descriptive praise *	A positive description of a child’s behavior or characteristic.
Specific instruction	A non-aversive instruction that implies the child should change his or her behavior. It is direct and has a clear behavioral referent.
Aversive specific instruction	An aversive instruction that implies the child should change his or her behavior. It is direct and has a clear behavioral referent.
Vague instruction	A non-aversive instruction that implies the child should change activity but is not direct or has no clear behavioral referent.
Aversive vague instruction	An aversive instruction that implies the child should change activity but is not direct or has no clear behavioral referent.
Question	Question or non-aversive request for information directed to the children.
Aversive question	Aversive question or request for information directed to the children.
Social attention	Parent gives positive (non-aversive) attention (verbal or non-verbal) to the child that cannot be coded elsewhere.
Aversive social attention	Parent gives negative (aversive) attention (verbal or non-verbal) to the child that cannot be coded elsewhere.
Performs side-by-side with the child *	Parent and child are both doing the puzzle. The parent does it at the same time as the child, not preventing them from participating (non-aversive).
Performs by Preventing the child from performing *	The parent in general prevents the child from performing, so only the parent performs the activity (aversive behavior).
Interruption	Interrupt the interaction with the child, between 2 and 5 s, to perform another activity in the time interval that this category is being completed.
No interaction	Absence of interaction, for more than 5 s, with the child when he is doing the activity.
*Child Behavior Codes*	
Compliance *	Compliance according to what is requested, that is, to obey a social instruction (verbal or nonverbal).
Noncompliance	Refuses to obey an instruction actively (verbalizing) or passively (ignoring).
Complain	Verbal complaints include whining, screaming, protesting, or outbursts of anger.
Aversive instruction	Aversive or unpleasant instructions directed at another person.
Aggression	Effective attacks or threats or hurting another person or destroying an object (e.g., punching, biting).
Opposing behavior	Other inappropriate behaviors that cannot be coded elsewhere.
Appropriate verbal interaction	Intelligible verbalizations of the child in the absence of any behavioral problems.
Involvement in the activity	Any appropriate behavior that does not include intelligible verbalizations (e.g., as following instructions, playing games, or responding to others).
Interruption	Any attempt to interrupt the parent’s activity or conversation with another person or to talk over the parent when they are talking to you.
No interaction	Absence of interactions with people or toys, repetitive manipulation of objects, or self-stimulation.

Note: All discrete behavior except those marked with * are described in FOS (Family Observation Schedule).

**Table 2:** The dimensions used in the global ratings.

Global Ratings	Description
Involvement	Active participation and cooperation of parents with a focus on the child’s activity.
Positive affectivity	A positive emotional expressiveness, the parent shows positive affection to the child, warmth in the relationship.
Directivity	Directive communication/action, preventing the child’s autonomous realization.
Responsiveness	Contingent response to the child’s needs when necessary, demonstrating appropriate emotional support.
Stimulus quality	Parental role using concepts/examples that allow the child to learn strategies to achieve the goal of the task, i.e., complete the jigsaw puzzle.

**Table 3:** Descriptive statistics and inter-rater agreement (ICC and κ) of the discrete behaviors and global ratings.

Discrete Behaviors	*Min–Max*	*M* (*SD*)	*Skewness*	*Kurtosis*	*ICC* (95% CI)
*Parent Behavior Codes*					
Praise	0.00–13.75	1.91 (2.99.00)	1.92	4.19	0.99 (0.97, 1.00) ***
Descriptive praise	0.00–4.62	0.40 (0.93)	2.86	9.21	0.92 (0.80, 0.97) ***
Specific instruction	1.03–27.50	12.80 (6.45)	0.27	−0.61	0.73 (0.39, 0.89) ***
Aversive specific instruction	0.00–11.25	1.89 (2.75)	1.90	3.29	0.68 (0.31, 0.87) ***
Vague instruction	0.00–18.00	7.91 (4.49)	0.55	−0.40	0.22 (−0.28, 0.62)
Aversive vague instruction	0.00–8.18	1.43 (2.26)	1.66	1.82	0.88 (0.70, 0.96) ***
Question	0.00–27.00	10.67 (6.70)	0.50	−0.24	0.48 (0.01, 0.77) *
Aversive question	0.00–5.00	0.44 (0.95)	3.05	11.38	0.97 (0.92, 0.99) ***
Social attention	0.00–30.00	7.46 (7.27)	1.43	1.55	0.89 (0.71, 0.96) ***
Aversive social attention	0.00–6.82	0.79 (1.64)	2.45	5.64	0.83 (0.59, 0.93) ***
Performs side-by-side with the child	0.00–30.00	24.75 (6.70)	−2.09	4.12	0.98 (0.95, 0.99) ***
Performs by preventing the child from performing	0.00–17.00	2.67 (3.20)	2.24	7.58	0.42 (−0.06, 0.74) *
Interruption	0.00–4.29	0.62 (0.98)	1.88	3.78	0.96 (0.90, 0.99) ***
No interaction	0.00–4.00	0.30 (0.74)	3.24	12.85	0.95 (0.86, 0.98) ***
*Child Behavior Codes*					
Compliance	0.00–3.00	0.34 (0.77)	2.36	4.92	0.54 (0.10, 0.81) **
Noncompliance	0.00–2.00	0.18 (0.50)	2.77	6.82	−0.05 (−0.51, 0.43)
Complain	0.00–8.57	1.11 (1.94)	2.46	6.19	0.74 (0.42, 0.90) ***
Aversive instruction	0.00–7.00	0.69 (1.50)	2.70	7.48	0.98 (0.94, 0.99) ***
Aggression	0.00–1.11	0.02 (0.16)	6.85	47.00	(a)
Opposing behavior	0.00–0.00	0.00 (0.00)	0.00	0.00	(a)
Appropriate verbal interaction	0.00–30.00	25.41 (7.46)	−2.47	5.55	0.99 (0.99, 1.00) ***
Involvement in the activity	26.79–30.00	29.72 (0.79)	−3.06	8.61	1.00 (0.99, 1.00) ***
Interruption	0.00–12.00	1.08 (2.21)	3.29	13.21	0.99 (0.98, 1.00) ***
No interaction	0.00–2.00	0.09 (0.35)	4.49	21.09	1.00 (1.00, 1.00) ***
Global ratings	*Min–Max*	*M* (*SD*)	*Skewness*	*Kurtosis*	*κ*
Involvement	0.00–4.00	1.66 (1.09)	0.73	−0.14	0.90 ***
Positive affectivity	0.00–4.00	1.09 (1.14)	0.84	−0.34	0.75 ***
Directivity	0.00–4.00	1.87 (1.08)	0.37	−0.56	0.72 ***
Responsiveness	0.00–4.00	1.70 (1.04)	0.40	−0.62	0.73 ***
Stimulus quality	0.00–4.00	1.40 (0.97)	1.03	0.83	0.74 ***

Note: (a) The standard deviations for both groups are 0 and, as such, the ICC could be computed. * *p* < 0.05, ** *p* < 0.01, and *** *p* < 0.001.

**Table 4:** Estimates and standard error for means and variances from the latent profile analysis.

	Class 1 (*n* = 35)	Class 2 (*n* = 12)
Means	*Estimate* (*SE*)	*Estimate* (*SE*)
Involvement	1.14 (0.11) ***	3.17 (0.27) ***
Positive affectivity	0.60 (0.14) ***	2.50 (0.36) ***
Responsiveness	1.26 (0.16) ***	3.00 (0.21) ***
Stimulus quality	0.97 (0.10) ***	2.67 (0.31) ***
Variances	*Estimate* (*SE*)	*Estimate* (*SE*)
Involvement	0.38 (0.08) ***	0.38 (0.08) ***
Positive affectivity	0.59 (0.15) ***	0.59 (0.15) ***
Responsiveness	0.48 (0.12) ***	0.48 (0.12) ***
Stimulus quality	0.38 (0.09) ***	0.39 (0.09) ***

Note: *** *p* < 0.001.

**Table 5:** Differences between classes in the discrete behaviors assessed by the micro-analytic coding procedure.

	Class 1 (*n* = 35)	Class 2 (*n* = 12)		
Behaviors	*M* (*SD*)	*M* (*SD*)	*t* (*df*)	*Cohen’s d* (95% *CI*)
*Parent Behavior Codes*				
Praise	1.01 (1.74)	4.53 (4.25)	−2.78 (12.29) *	−1.36 (−2.06, −0.64)
Descriptive praise	0.33 (0.91)	0.60 (1.01)	−0.86 (45)	−0.29 (−0.95, 0.37)
Specific instruction	11.15 (5.63)	17.64 (6.46)	−3.32 (45) **	−1.11 (−1.80, −0.41)
Aversive specific instruction	2.27 (3.01)	0.78 (1.41)	1.65 (45)	0.55 (−0.12, 1.21)
Vague instruction	6.98 (3.82)	10.61 (5.34)	−2.55 (45) *	−0.85 (−1.53, −0.17)
Aversive vague instruction	1.74 (2.51)	0.52 (0.82)	2.51 (44.98) *	0.55 (−0.12, 1.21)
Question	8.52 (5.13)	16.94 (6.97)	−4.47 (45) ***	−1.49 (−2.21, −0.76)
Social attention	5.59 (5.97)	12.92 (8.21)	−3.33 (45) **	−1.11 (−1.80, −0.41)
Aversive social attention	1.03 (1.84)	0.08 (0.29)	2.94 (38.45) **	0.59 (−0.08, 1.25)
Performs side-by-side with the child	26.88 (3.42)	18.52 (9.75)	2.91 (11.94) *	1.58 (0.75, 2.19)
Performs by preventing the child from performing	3.05 (3.47)	1.55 (1.98)	1.42 (45)	0.47 (−0.19, 1.13)
Interruption	0.60 (0.99)	0.69 (1.00)	−0.28 (45)	−0.09 (−0.75, 0.56)
*Child Behavior Codes*				
Compliance	0.19 (0.55)	0.76 (1.14)	−1.65 (12.80)	−0.77 (−1.44, 0.09)
Noncompliance	0.10 (0.32)	0.43 (0.80)	−1.39 (12.24)	−0.68 (−1.35, −0.01)
Complain	1.34 (2.16)	0.42 (0.79)	1.44 (45)	0.48 (−0.18, 1.14)
Aversive instruction	0.70 (1.54)	0.67 (1.44)	0.06 (45)	0.02 (−0.63, 0.68)
Appropriate verbal interaction	25.55 (7.19)	25.02 (8.54)	0.21 (45)	0.07 (−0.59, 0.73)
Involvement in the activity	29.65 (0.89)	29.92 (0.29)	−1.54 (44.97)	−0.34 (−0.99, 0.32)

**Note:** (a) The standard deviations for both groups are 0 and, as such, the *t*-test could be computed. * *p* < 0.05, ** *p* < 0.01, and *** *p* < 0.001.

**Table 6:** Differences between classes on the measured outcome variables.

	Class 1 (*n* = 35)	Class 2 (*n* = 12)		
Measures	*M* (*SD*)	*M* (*SD*)	*t* (*df*)	*Cohen´s d* (95% *CI*)
*Affection Scale*				
Communication-affection	30.63 (4.22)	31.08 (3.32)	−0.34 (45)	−0.11 (−0.77, 0.54)
Criticism-rejection	8.20 (3.39)	9.00 (3.54)	−0.70 (45)	−0.23 (−0.89, 0.43)
*Alabama Parenting Questionnaire*				
Positive parenting	46.09 (6.29)	48.55 (4.66)	−1.19 (44)	−0.41 (−1.09, 0.27)
Poor monitoring	7.77 (4.17)	8.36 (4.13)	−0.41 (44)	−0.14 (−0.82, 0.54)
*Parental Stress Index*	119.57 (26.80)	128.00 (18.21)	−1.93 (43)	−0.33 (−1.04, 0.37)

**Table 7:** Differences between classes on the continuous sociodemographic variables.

	Class 1 (*n* = 35)	Class 2 (*n* = 12)		
Measures	*M* (*SD*)	*M* (*SD*)	*t* (*df*)	*Cohen´s d* (95% *CI*)
*Parents’*				
Age	36.23 (7.17)	35.00 (7.05)	0.51(45)	0.17 (−0.49, 0.83)
Number of children	2.82 (1.16)	2.42 (1.17)	0.99 (45)	0.33 (−0.33, 0.99)
Years of schooling	8.23 (3.50)	6.08 (2.68)	1.93 (45)	0.65 (−0.03, 1.31)
*Children’s*				
Age	9.00 (1.23)	8.67 (1.67)	0.53 (45)	0.18 (−0.48, 0.83)
Years of schooling	3.54 (1.76)	3.42 (1.83)	0.07 (45)	0.72 (−0.59, 0.73)

## Data Availability

Data supporting reported results are available at osf.io/guxk5 (accessed on 4 October 2021).
